# Quality Parameters of Wort Produced with Lentil Malt with the Use of Some Enzymatic Preparations

**DOI:** 10.3390/foods14050848

**Published:** 2025-03-01

**Authors:** Katarzyna Fulara, Aneta Ciosek, Olga Hrabia, Monika Cioch-Skoneczny, Krystian Klimczak, Aleksander Poreda

**Affiliations:** Department of Fermentation Technology and Microbiology, Faculty of Food Technology, University of Agriculture in Krakow, Balicka 122, 30-149 Krakow, Poland; aneta.ciosek@urk.edu.pl (A.C.); o.hrabia@urk.edu.pl (O.H.); monika.cioch@urk.edu.pl (M.C.-S.); krystian.klimczak.sd@student.urk.edu.pl (K.K.); a.poreda@ur.krakow.pl (A.P.)

**Keywords:** lentil, lentil malt, congress mash, novel raw material

## Abstract

Lentils represent a promising alternative for beer production, potentially offering unique benefits and challenges. This study investigates the physicochemical properties of brewer’s wort derived from both barley and lentil grains. Specifically, it compares worts produced from raw and malted lentils, with and without the addition of amylase and protease enzymes. Key parameters such as filtration and saccharification times, pH, extract content, color, turbidity, polyphenol content, free amino nitrogen (FAN), nitrogen content, and metal ion and sugar composition were meticulously measured. Results indicate that both raw and malted lentils can be utilized to produce brewer’s wort, with the malting process enhancing extract levels. Notably, the addition of amylolytic enzymes resulted in the highest extract levels for both lentil types. Lentil-based worts exhibited significantly higher FAN levels and lower turbidity compared to barley malt worts. Despite barley malt’s established advantages in saccharification efficiency, filtration, and extract yield, lentils offer distinct benefits such as elevated FAN levels and unique color profiles. Enzyme treatments play a crucial role in optimizing lentil-based wort production, highlighting the potential for lentils in brewing applications.

## 1. Introduction

For many years, barley malt has been the cornerstone of brewing. However, to mitigate costs, breweries often incorporate adjuncts such as corn and rice. The search for alternative raw materials has intensified due to rising ingredient prices, economic inflation, and occasional shortages of barley malt. Moreover, using alternative components can impart unique sensory characteristics to the final product. Globally, approximately 85–90% of beer is produced with the use of unmalted raw materials [1]. Research has identified several potential alternatives, including cereals like Teff (*Eragrostis tef*) and pseudo-cereals such as Amaranth (*Amaranthus* spp.) and quinoa (*Chenopodium quinoa*) [2,3,4]. Additionally, legumes are emerging as a promising option for the brewing industry due to their beneficial properties.

Lentils (*Lens culinaris*) are one of the oldest human-cultivated crops, along with wheat and barley. The first traces of its use date back to 11,000 BC [5]. Although for some time the plant was forgotten, and considered as food for the poor, in recent years it has gained popularity in various industries. According to the FAO, its production has more than doubled in the last 30 years, reaching 7.6 million tons in 2017 [6]. Compared to barley, grains of lentils are characterized by a lower carbohydrate content (78–83% and 43.4–69.9%, respectively). In the carbohydrate fraction, starch (35–63 g/100 g d.m.), galacto-oligosaccharides (2–8 g/100 g d.m.), simple sugars (1–2.5 g/100 g d.m.), and non-starch polysaccharides (~20 g/100 g d.m.) are distinguishable. Lentils differ from barley in that they have a higher protein content (20.6–31.4%) and no gluten, making them suitable for people with celiac disease or gluten intolerance. The development of gluten-free beer products is becoming increasingly important [5,7].

Recent studies support the feasibility of using lentils in brewing. Trummer et al. [8] and Gasiński & Kawa-Rygielska [9] have shown that malted lentils can enhance foam stability, reduce gluten content, and provide unique flavor profiles in beer. However, challenges such as attenuation and filtration times remain and warrant further optimization. The high protein and nitrogen content in lentils contribute significantly to wort quality, which could make them a valuable adjunct for brewing specialty and gluten-free beers.

Incorporating lentils into the brewing industry offers numerous health, environmental, and economic benefits. Currently, methods such as enzymatic reactions and precipitation are used to remove gluten from beer; however, these techniques are not foolproof and may leave residual gluten in the final product. Safer alternatives include using inherently gluten-free raw materials such as rice, oats, maize, sorghum, and teff. Given that lentils are naturally gluten-free, they present a promising solution for producing gluten-free beer [5,10]. Lentils contain a relatively high level of zinc, ranging from 3.2 to 6.3 mg per 100 g. Zinc is an essential element in beer production, playing a crucial role in maintaining yeast health. In comparison, barley contains zinc levels ranging from 0.6 to 7.1 mg per 100 g. Therefore, worts made from lentils have the potential to have a higher zinc content compared to worts made from barley. Lentils also contain considerable amounts of magnesium (122 mg/100 g d.m.) [11,12]. The concentration of magnesium ions in the wort is an important parameter affecting the viability of yeast cells and the process of effective fermentation. Magnesium is a cofactor for over 300 enzymes, including amyloglucosidase and alkaline phosphatase, which are responsible for the breakdown of starch and proteins during mashing. The appropriate level of magnesium in the wort affects the effectiveness of these enzymes, which translates into the efficiency of the beer brewing process. Lentils possess relatively modest cultivation requirements, which renders them suitable for growth in varied regions of the world. Like other pulses, lentils exhibit nitrogen fixation ability, which helps to augment soil fertility naturally. The expansion of legume acreage may aid in minimizing the use of synthetic fertilizers [13].

The aim of this research was to produce brewer’s wort using malted green lentils and analyze its biochemical properties. This study also explored unmalted lentils to compare the impact of malting on wort quality. Additionally, amylolytic and proteolytic enzymes were tested during mashing to evaluate their effectiveness in improving lentil-based worts. Barley malt served as the control. Parameters such as saccharification, filtration time, pH, color, extract content, nitrogen content, and free amino nitrogen (FAN) were assessed, along with the carbohydrate profile and metal ion concentrations.

## 2. Materials and Methods

### 2.1. Materials

Green lentils (*Lens culinaris*) from Canada and barley malt pils type (IREKS, Kulmbach, Germany) were used in this study. Two enzyme preparations were used: Maxazyme NNP DS (MAX), a bacterial protease with proteolytic activity, and Mats L Classic (MLC), a bacterial thermostable α-amylase (both from DSM Food Specialties, Delft, The Netherlands). Enzyme preparations were added at the start of congress mashing at the highest dosage recommended by the producer, which equaled 2 g of MAX and MLC per 1 kg of grain.

### 2.2. Malting Procedure

Steeping and germination of the lentils were performed in an aerated cabinet Q-Cell 240 (Pol-Lab Sp. K., Wilkowice, Poland). For drying and kilning, a Memmert SLL 400 drying oven (Memmert GmbH & Co. KG, Schwabach, Germany) was used. The steeping process was carried out at 20 °C for 16 h, and germination was performed at the same temperature for 48 h, with drying and kilning at 50 °C for five hours and 120 °C for three hours. The moisture content of the lentil malt was 2.34 *w*/*w* d.m. The lentils were frequently mixed during germination and kilning. Moisture content after kilning was measured with a Radwag MAC 50 moisture analyzer (Radwag, Radom, Poland).

### 2.3. Mashing—Congress Wort Production

All samples were mashed in an R12 mash bath (1-CUBE s.r.o., Prague, Czech Republic) using the EBC method 4.5.1 Extract of Malt: Congress Mash (Analytica EBC, 2004). For this purpose, 50 g of milled malt (barley and lentil) and lentil grains were weighed and placed in mash containers. 200 mL of distilled water at 45 °C was gradually added to the containers. 0.1 g of MAX and 0.1 g of MLC were added to selected trials. The mash bath was held at 45 °C for 30 min, then the temperature was raised at a rate of 1 °C/min until reaching 70 °C. Once the apparatus reached 70 °C, an additional 100 mL of distilled water (70 °C) was added to the containers. Mashing continued for 1 h. Afterward, the containers were cooled to 20 °C and filled up with distilled water to reach 450 g, and filtered through a paper filter. To ensure high clarity, the first portions of the filtrate were recirculated. The filtered wort was collected for analyses.

### 2.4. Analytical Method

#### 2.4.1. Saccharification Time

Saccharification time was assessed according by the Analytica EBC 4.5.1 method [14]. After adding water at 70 °C to the mashes, the measurement of saccharification time sbegan, and the iodine test was performed at 5-min intervals. If the iodine solution did not turn blue, full starch saccharification was considered complete, and the saccharification time was recorded. The final test was conducted after 60 min of mashing at 70 °C.

#### 2.4.2. Filtration Time

Filtration was performed by the Analytica EBC 4.5.1 method [14]. Before starting with the filtration, the samples were mixed thoroughly with a glass rod and then poured into a funnel with filter paper. The filtrate was collected in 500 mL graduated cylinders. The first 100 mL of filtrate was returned to the funnel and then the filtration timer started. The filtration was finished when the filtration cake appeared dry.

#### 2.4.3. Determination of Wort pH

Wort pH was measured using a CP-411 pH-meter (Elmetron SP. J., Zabrze, Poland). Before analysis, the pH-meter was calibrated with buffer solutions (pH 4.0 and 7.0).

#### 2.4.4. Determination of Color

The color of the wort was determined spectrophotometrically using a Beckman DU-650 UV-Vis spectrophotometer (Beckman Coulter Inc., Brea, CA, USA) at a wavelength of 430 nm, according to Analytica EBC Methods 8.5 [14].

#### 2.4.5. Determination of Wort Extract Content

The extract content of the wort was assessed using a DMA 35 densimeter (Anton Paar GmbH, Graz, Austria).

#### 2.4.6. Turbidity

The turbidity of the wort was analyzed using a Eutech TN 100 turbidity meter (Fisher Scientific GmbH, Schwerte, Germany).

#### 2.4.7. Determination of Polyphenols

The Total Polyphenol content in wort was measured using a Beckman DU-650 UV-Vis spectrophotometer, according to the EBC method 8.12 [14]. Samples of wort (10 mL) were mixed with 8 mL of CMC/EDTA reagent (10 g/L sodium carboxymethyl cellulose and 2 g/L disodium EDTA), 0.5 mL of ferric reagent (5.6 g/L ferric ions), and 0.5 mL of ammonia reagent (diluted 1:3 with water) in 25 mL volumetric flasks. The final volume was adjusted to 25 mL with distilled water.

After mixing, the reaction was allowed to proceed for 10 min at room temperature, and the absorbance was measured at 600 nm using a Beckman DU-650 UV-Vis spectrophotometer. All measurements were performed in triplicate, with reagent blanks used to correct for background absorbance.

#### 2.4.8. Determination of Nitrogen

Total nitrogen content in wort was determined using the Kjeldahl method according to EBC method 8.9.1 [14]. Approximately 1.0 to 1.5 g of the wort sample was mixed with about 10 g of a catalyst mixture (1000 parts m/m potassium sulfate, 30 parts m/m titanium dioxide, and 30 parts m/m copper (II) sulfate pentahydrate) in a Kjeldahl flask. Then, 20 mL of 98% sulfuric acid was added, and the mixture was digested until a clear solution was obtained.

The digest was diluted with 250 mL of distilled water and made alkaline with 70 mL of sodium hydroxide solution (450 g of NaOH in 1 L of water). Released ammonia was distilled into a 20 g/L boric acid solution containing 0.5 mL of a mixed indicator (bromocresol green and methyl red). The ammonia was then titrated with 0.1 mol/L hydrochloric acid or 0.05 mol/L sulfuric acid to the endpoint.

#### 2.4.9. Determination of Free Amino Nitrogen Content

Free amino nitrogen (FAN) content in wort was measured using a ninhydrin-based spectrophotometric method at 570 nm, as described in EBC method 8.10 [14]. Diluted wort samples (1:100 with distilled water) were mixed with 1 mL of ninhydrin reagent, containing Na_2_HPO_4_, KH_2_PO_4_, ninhydrin, and fructose, and heated in a boiling water bath for 16 min. After cooling to 20 °C, 5 mL of diluting solution (KIO_3_ in ethanol) was added. The absorbance was measured at 570 nm using a Beckman DU-650 UV-Vis spectrophotometer. For dark worts, a color correction was applied. Glycine standards were used for calibration, and FAN was expressed in mg/L.

#### 2.4.10. Analysis of Metal Ion Content

Metal ion concentrations of wort were determined using atomic absorption spectrometry with flame atomization using a Varian AA240FS spectrophotometer (Varian Inc., Palo Alto, CA, USA) equipped with an automatic sample-dispensing system (SIPS-20, Varian Inc., Palo Alto, CA, USA). Wort samples (3 mL) or grain samples (0.3 g) were placed in sealed pressure vessels with 5 mL of nitric acid (68%) and subjected to wet mineralization in a Mars Xpress microwave oven (1200 W, 170 °C, 15 min) (CEM Corp., Matthews, NC, USA). The digested samples were then diluted with deionized water, and absorbance was measured at 202.6 nm for Mg^2+^, 422.7 nm for Ca^2+^, and 213.9 nm for Zn^2+^. The gas flow rates were 3.5 L/min for acetylene and 14 L/min for air. Standard solutions for Mg^2+^, Ca^2+^, Zn^2+^ (100, 40 and 5 mg/L, respectively) were prepared from a 1000 mg/L standard solution (Merck KGaA, Darmstadt, Germany).

#### 2.4.11. Carbohydrate Profile Analysis

The sugar content was analyzed using Reversed-Phase High-Performance Liquid Chromatography (RP-HPLC). The analysis was performed at 30 °C using a Dionex UltiMate 3000 system equipped with a refractometric detector (RefractoMax520, Thermo Scientific, Germering, Germany).

Samples were prepared by dilution with deionized water and filtered through a 0.45 µm filter before injection. A 20 µL volume of each sample was injected into a Kromasil RP 100-5 NH_2_ column (250 × 4.6 mm). The mobile phase consisted of a mixture of acetonitrile and water (80:20 *v*/*v*) at a flow rate of 1.3 mL/min. The elution was monitored by measuring the refractive index at the detector. Calibration was performed using standard sugar solutions to ensure the accuracy and precision of the measurements.

### 2.5. Statistical Analysis

The results are presented as the mean of three independent experiments, with error bars representing the standard deviation. Data were analyzed using one-way and multivariate analysis of variance (ANOVA). The significance of differences for each parameter was assessed separately, using Tukey’s post hoc test (Statistica v. 10, StatSoft Inc., Kraków, Poland).

## 3. Results and Discussion

### 3.1. Physicochemical Parameters of the Congress Worts

#### 3.1.1. Saccharification Time

For the wort made from barley malt, saccharification occurred within the expected range of 5 to 10 min [15]. In contrast, neither raw nor malted lentils samples saccharified during 1 h of mashing at 70 °C (Table 1). The low enzymatic activity of lentils, caused by the presence of amylase inhibitors, may prevent complete saccharification of the mash [16,17]. Additionally, during malting, lentil grains were dried at a higher temperature (120 °C) compared to barley malt (80 °C). This higher temperature may have led to reduced enzymatic activity, as elevated temperatures generally impair enzyme function. The choice of 120 °C kilning temperature was based on previous studies [18], which showed that lower kilning temperatures led to poor sensory quality. Thus, the higher kilning temperature was justified, as it enhanced malt aroma (distinctive, with reduced pea-like notes) and deepened color, despite the decline in enzymatic activity.

Considering that temperature can affect enzyme activity, lentils require higher temperatures during malting to activate specific enzymes such as amylases and proteases, which are crucial for starch degradation and protein modification. These enzymatic activities are essential for producing fermentable sugars and improving the overall quality of the wort. Previous studies indicate that lentil enzymes exhibit different optimal temperature profiles compared to barley, thus supporting effective wort production [18].

Additionally, lentils have unique moisture absorption characteristics, necessitating careful control of steeping and drying temperatures. The initial steeping at 20 °C, followed by drying at 50 °C and kilning at 120 °C, helps to maintain optimal moisture levels, prevent microbial growth, and ensure uniform germination. The higher kilning temperature (120 °C) is particularly important for developing the flavor profile of lentil malt, as it promotes Maillard reactions and caramelization, enhancing the sweetness and complexity of the final product. However, it is essential to monitor these temperatures closely to avoid denaturing enzymes critical for subsequent brewing processes. Given the higher protein content of lentils, adjustments in the malting process were necessary to optimize enzymatic functionality and enhance the sensory attributes of the resulting malt.

In the wort made from malted lentils, with the addition of an enzyme preparation containing α-amylase activity, saccharification occurred after 25 min. Previous studies [19], reported similar saccharification times (25 min) for lentil malt with the same enzyme preparation. For the mash made from raw lentil grains with the addition of α-amylase (MLC), saccharification occurred after 40 min of mashing. Thus, the use of an enzyme preparation that facilitates starch hydrolysis is essential for efficient lentil wort production. Moreover, using malted lentils reduced the saccharification time by approximately 15 min. One method that may expedite starch hydrolysis is starch gelatinization [19], which increases the accessibility of starch for amylolytic enzymes, leading to faster and more effective saccharification [20]. The addition of protease to both malted and raw lentils did not result in saccharification.

#### 3.1.2. Filtration Time

The filtration time of the reference sample (BM), made from barley malt, was the shortest at 10.7 min. Similarly times were short for the sample with the addition of α-amylase, whereas the addition of protease resulted in prolonged filtration times. Filtration time for congress wort, typically taking less than one hour, are generally considered to be within the normal range. Therefore, filtration times for all barley malt samples in this study can be regarded as normal. The filtration time of malted lentils was 80 min, and the addition of amylase and protease reduced this time to 64 and 42.7 min, respectively. Notably, the filtration time for raw lentil grain was shorter, at 50 min. The addition of amylase further reduced this time to 25.3 min. Conversely, protease addition significantly increased filtration time to 95.7 min. This may be due to the formation of protein breakdown products, which can increase the viscosity of the wort.

Several factors influence the filtration process, including the quality of the raw material, homogeneity, particle size distribution, the degree of degradation of high molecular weight polysaccharides (particularly non-starch ones, such as β-glucans and arabinoxylans), and the presence of proteins sensitive to oxidation. 

Lentils contain fewer glucans (0.3% d.m.) compared to barley (3.6–9.0% d.m.) [21], which could theoretically be beneficial for filtration speed. However, such a relationship was not observed in the experiment. In all wort samples made from green lentil grain, the formation of a gel layer (Oberteig’s layer) was observed on top of the filter bed. This gel layer is a complex of macromolecules, including proteins, lipids and polysaccharides derived from the cell wall of the grain [22]. It was observed that filtration rate decreased as the gel layer consolidated, leading to a blockage of the filter. This layer is composed of very fine particles and has clay-like properties in both appearance and consistency. Similar layers are also observed during the formation of spent grains when malted barley varieties with exceptionally high protein content are used. Brewers generally avoid high-protein barley varieties, as they are associated with prolonged wort filtration times [23,24].

In both lentil variants, the addition of amylolytic enzymes reduced the filtration time. The different effects of α-amylase and protease on filtration time can be attributed to the distinct mechanisms by which these enzymes act on the lentil grains. α-Amylase hydrolyzes starch into smaller polysaccharides and simple sugars, thereby reducing the viscosity of the wort. Although raw lentils have a lower starch content compared to barley, they still contain sufficient starch for α-amylase to exert a significant effect on filtration time. The addition of α-amylase to raw lentils breaks down the starch, facilitating easier flow during filtration, which explains the substantial reduction in filtration time observed in these samples.

In contrast, protease acts on proteins, breaking them down into smaller peptides and amino acids. Proteins in the lentil grains can form aggregates or complexes that contribute to higher viscosity and slower filtration. The presence of such protein aggregates can impede filtration, especially in raw lentils, where protease may increase the number of small particles in the wort, leading to increased viscosity and slower filtration. However, in malted lentils, where some of the protein aggregates have already been degraded during malting, the addition of protease likely further breaks down these aggregates, leading to improved filtration efficiency and a reduction in filtration time. Therefore, while α-amylase consistently reduced filtration time in both raw and malted lentils by breaking down starch, protease had contrasting effects depending on the initial protein structure in the grains. This difference in enzyme activity highlights the complex interactions between starch, protein content, and enzyme action during the filtration process of lentil grains. Specifically, protease may increase filtration time in raw lentils by promoting the formation of additional particles, whereas it can enhance filtration efficiency in malted lentils by further breaking down protein aggregates.

#### 3.1.3. Wort pH

The pH of the mash plays a crucial role in determining several key factors of the brewing process, including the concentration of the extract, the solubility of hop components, and the color of the wort [25]. The pH values of the analyzed worts ranged from 5.9 to 6.4. The wort produced from malted lentils was characterized by a higher pH (pH 6.05) compared to the control sample made from pilsner malt (pH 5.89). Notably, the addition of amylolytic and proteolytic enzymes to the lentil mashes did not affect the pH. The highest pH values were determined in the sample of wort produced from green lentil grain (pH 6.41).

The pH of the wort should not exceed the value of 6.0, as maintaining pH within a specific range is crucial for optimizing enzymatic activity and preventing the extraction of undesirable compounds like polyphenols and tannins, which can contribute to astringency and off-flavors in the beer [25,26,27]. The worts produced from both raw and malted lentils in this study exceeded this threshold, with previous research showing similar high pH ranges for lentil-based worts, from 6.0–6.2 in malted, and 6.3–6.4 in raw lentil worts [28].

Higher wort pH can also impact other important brewing parameters. A pH above 6.0 can inhibit the formation of hot trub during boiling, leading to poorer colloidal stability in the finished beer [27,29]. Conversely, a pH in the optimal range of 5.2–5.6 is favorable for enzyme activities like α-amylase, which drive efficient starch saccharification and protein breakdown, resulting in better extraction of fermentable sugars and nutrients from the grain [30]. It also enhances yeast fermentation performance and improves wort clarification [31].

The elevated pH values observed in lentil-based worts can be corrected by adjusting the brewing water, using acidifying malts, or directly acidifying the wort with biological or mineral acids [32]. This step would help align the lentil wort’s pH with that of traditional barley-based worts, improving brewing efficiency and final product stability.

#### 3.1.4. Color

The color of the wort results from melanoidins, i.e., color compounds that are formed during the kilning process (Maillard reaction between amino acids and reducing sugars) [33,34].

The color of the congress wort made from barley malt was 5 EBC. The addition of α-amylase did not influence the color of the barley malt wort. However, the color was slightly deepened when protease was used. This is probably due to the higher FAN content of protease-treated wort. No significant changes were seen in the case of raw lentils worts. The worts obtained from raw lentils were characterized by higher color values than those obtained from barley malt, but the enzymes did not have any significant effect on the color of these worts. On the contrary, changes were observed for the worts obtained with malted lentils. The base wort produced with malted lentils was characterized by a much darker color (38 EBC) than previously discussed variants. Additionally, both enzyme additions resulted in the darkening of the wort (Table 1). The wort obtained with the addition of protease was characterized by the darker color of all the samples (44 EBC). The darker color of that sample, compared to the sample without enzyme addition, might be due to the higher FAN content of that sample.

Overall, the darker color of worts obtained from malted lentils is probably due to the Maillard reactions which took place during the grain drying process (malting stage) and higher FAN content. In the experiment, malted lentil grain was dried using higher temperatures compared to the standard barley malt production process (120 °C and 80 °C, respectively), which may be related to obtaining a darker color of the wort. In addition, an almost two times higher concentration of FAN in malted lentil wort could have resulted in a more intense color of the wort. By modifying the temperature and duration of the drying step during the malting process, the malt can take on different color intensities, which is a key determinant of the appearance of the wort and beer. A common practice used in breweries to deepen the color of the wort is the addition of dark malts to the grist, or just obtaining a higher FAN concentration of the wort by adding commercial proteolytic enzymes, such as MAX [35]. Another reason for the higher color of lentil malt wort can be the extent of non-enzymatic browning reactions (Maillard reaction) and the extraction of color-active substances from the raw materials which is greater at a higher pH value [25,34,36].

#### 3.1.5. Extract

The extract content in the congress wort produced from barley malt was 9.0 °P. The addition of enzymes did not significantly change the extract levels of barley malt worts. The wort made from malted lentils (ML) was characterized by a lower extract concentration of 3.4 °P. The addition of an amylolytic enzyme (MLC) resulted in an almost twofold increase of extract content both for malted and raw lentils worts (6.7 °P and 6.5 °P, respectively). Interestingly, the addition of protease to raw lentils allowed to obtain slightly higher extract levels (Table 1). The results for the samples with amylase addition show that if full saccharification occurred, lentils produce about 2/3 of the extract as compared to barley. Such results are in line with the literature data regarding sugar content in lentils [5]. In the case of barley grain, the malting process generates sufficient enzymatic potential to fully saccharify the sugars contained in a grain. This is confirmed by the short saccharification time and no significant effect of enzyme addition on the resulting extract (Table 1). In the case of lentil malt, the malting process of lentils does allow to obtain a wort with a higher extract content than raw grain, but the enzymatic power is not high enough to convert all potential extract into soluble matter. Exogenous amylase enzymes seem crucial to obtain the extract from lentils, regardless of whether it is in a raw or a malted form.

#### 3.1.6. Turbidity

The wort produced with malted barley was characterized by the highest turbidity across the samples (84 NTU). The addition of both α-amylase and protease resulted in less turbid wort (Table 1). The wort obtained from raw lentils without enzyme additions was characterized by far higher turbidity compared to malted lentils (41 and 16.4 NTU, respectively). However, when enzymes were employed in raw lentil variants a significant reduction in turbidity was observed (6.0 and 5.9 NTU, respectively). Overall, these samples were characterized by the lowest turbidity across the variants. There was no significant change of turbidity when enzymes were used in malted lentil samples. The obtained results are similar to the results obtained by other researchers [8], where a higher number of lentils in a grain bill resulted in lower turbidity levels of a resulting wort. Some malts can give excessively turbid worts as a result of improper harvest, storage or malting conditions, which might give an opportunity for some microbial species to develop [37]. In the study, the addition of α-amylase allowed to significantly reduce wort turbidity. The same results can be seen in this study (Table 1). Higher lauter turbidity is known to positively influence yeast metabolism and performance, but on the other hand, it has detrimental effects on final beer quality, especially on flavor. Higher turbidity worts are recognized for producing more trub and causing extract losses due to the difficulty in separating it. As a result, low turbidity worts are often desired [38].

#### 3.1.7. Polyphenols

Table 1 shows polyphenol content in worts obtained with the use of various grists. In the reference wort (made using barley malt), 86.5 mg/L of polyphenols was determined. Malted lentils wort contained a higher number of polyphenols, ca. 125 mg/L. In the raw green lentil wort (RL), 188 mg/L of polyphenols was determined. This was significantly higher compared to wort produced from malted lentils. Polyphenol concentration was over two times higher compared to the control wort (BM 86.5 mg/L). The addition of α-amylase and protease did not significantly change the concentration of polyphenols for both malted barley and lentils. On the contrary, significant changes were observed in raw lentils variants. The addition of α-amylase resulted in elevated levels of these compounds, but even higher concentrations were obtained in a sample with the addition of protease, where 285 mg/L of polyphenols was detected.

We observed a very strong effect of malting on the concentration of polyphenols in lentils. In all samples, the malting process caused a significant decrease in polyphenols content in the resulting wort. It is likely that the steeping process and removal of rootlets resulted in a lower number of polyphenols in the grains. This behavior is in contrast to that reported for barley malting. Several authors have reported that in the case of barley, malting causes an increase in the polyphenol content of the grain and the resulting wort [39,40].

Polyphenols can enhance the sensory stability of beer by acting as antioxidants, but they also influence characteristics such as color, astringency, foam stability, and turbidity. Excessive levels of polyphenols may negatively impact the sensory qualities of beer, as they are believed to contribute to undesirable bitterness and haze formation. As the main natural antioxidants in brewing raw materials and beer, polyphenols play a crucial role in preserving freshness and preventing oxidation [41,42].

#### 3.1.8. Nitrogen

The importance of nitrogenous substances in the production of beer and its quality is very significant. Growing conditions have the greatest influence on the total content of nitrogenous substances in raw grain of barley. It is also affected by the variety of barley and malting technology. Only some of the total nitrogen passes from the malt into the wort. The nitrogen content of wort affects the technological process and the sensory properties of beer. Soluble nitrogenous usually ranges from 650 to 950 mg/L of wort [43].

Table 1 displays the total nitrogen content in congress worts produced from various raw materials. The reference wort made from barley malt contained 693 mg/L of total nitrogen. In comparison, the wort produced from malted lentils had a significantly higher nitrogen content, measuring 1557 mg/L. This is consistent with the higher protein content in lentils, which is approximately 25% d.m., more than double the protein content of barley, which ranges from 10–12% d.m. As expected, the wort made from malted lentils contained roughly twice the amount of nitrogen compared to the wort made from barley malt.

The wort produced from raw lentils contained 1219 mg/L of nitrogen, which is lower than that in wort from malted lentils but still considerably higher than the nitrogen content of the barley malt wort. The amount of protein degradation products released into the water depends on their amount in the raw material, as well as on the level of activity of proteolytic enzymes. The malting process increased the activity of proteolytic enzymes. In the case of a wort made from raw lentils with amylolytic activity (MLC) addition, the protein content remained at the same level as in the wort made from raw lentils. On the contrary, adding an enzyme with proteolytic activity (MAX) to the raw lentil mash led to an increased content of nitrogen in the wort by 30% (RL + protease = 1692). Congress wort with the highest amount of dissolved protein (2446 mg/L total nitrogen) was obtained with the use of malted lentils and the addition of proteolytic enzyme.

Protein concentration is an important parameter that needs to be monitored during wort production. Too high a level of protein is undesirable due to the occurrence of haze responsible for the deterioration of the colloidal stability of the beer and beer aroma (higher alcohol production). The above results lead to the conclusion that the proteolytic enzymes present in green lentils are at the appropriate level and there is no need for their additional supplementation in order to not exceed the desired level of total and soluble nitrogen in wort and beer.

#### 3.1.9. Free Amino Nitrogen

Free Amino Nitrogen (FAN) includes low molecular weight protein compounds present in the wort which are essential for healthy yeast growth and development. Optimal levels of FAN vary and depend on the type of fermentation performed, as well as yeast strain and the concentration of reducing sugars [44]. FAN concentrations in the range of 200–240 mg/L are considered optimal for efficient fermentation of the wort with an extract concentration of 10–12 °P [45,46,47]. It is assumed that the minimum content of FAN required to achieve satisfactory yeast growth and ensure proper fermentation efficiency in a wort with an extract of 10–12 °P is about 130 mg FAN/L [46,48]. Below this concentration, yeast growth is nitrogen dependent and suboptimal available nitrogen concentrations are associated with retardation, incomplete fermentation and sulfide release [46,49].

The lowest content of free amino nitrogen was determined in the wort produced from barley malt (157 mg/L). This wort was characterized by an extract content of 9 °P, so it can be assumed the FAN level in standard wort of 12 °P would reach ca. 210 mg/L, which is right in the optimal range.

The wort made from malted green lentils had almost twice as much free amino nitrogen (311 mg/L) as the wort made from barley malt. This is because green lentils contain twice as much protein in their composition (25% d.m.), compared to barley grains (10–12% d.m.) of which a substantial part is soluble (Table 1). The use of raw lentils resulted in a wort with a higher FAN level (172 mg/L) compared to barley malt, but not as high as with malted lentils. The higher concentration of FAN when using lentil malt compared to raw grain is due to the fact that some of the available proteins naturally present in the grain have already been partially hydrolyzed by endogenous proteolytic enzymes, activated during the malting process [50].

The addition of a product with α-amylase activity (MLC) to the mash resulted in slightly lower FAN levels in all the samples (Table 1). The addition of proteolytic enzyme, predictably, resulted in elevated FAN levels in all the samples. The FAN content of the barley malt variant was right in the optimal range for efficient fermentation. The FAN levels of lentils samples were above 300 mg/L (Table 1). This suggests that exogenous proteolytic enzymes were able to break down larger peptides into smaller ones to provide yeast cells with more available assimilable nitrogen sources. Therefore, the highest content of FAN was determined in the sample of wort produced from malted green lentils with the addition of a proteolytic enzyme (352 mg/L). It was more than two times higher as compared to wort samples obtained from barley malt.

Wort made from raw green lentils compared to worts made from other grains used in brewing (barley, wheat, oats, rye), allowed to obtain a much higher content (RL = 172 mg/L) of free amino nitrogen compared to these cereals (respectively 63.2; 90.8; 78.9; 85.2 mg/L) [35]. These results are very important from a practical perspective. On one hand, too high a content of FAN in the pitching wort can lead to sensory issues (mainly increased diacetyl production). Therefore, the usage of lentil malt will take this quality parameter into account when recipes are formulated. On the other hand, worts are often deficient in FAN, so the application of some lentil malt in the grist can be a good solution for increasing FAN content.

#### 3.1.10. Metal Ion Content

Certain metal ions are crucial in the brewing process. Calcium plays a crucial role throughout the process, stabilizing enzymes during mashing, promotes the lowering pH of the mash, and enhancing protein precipitation in the boil. Calcium ions stabilize α-amylase during mashing, protecting it against high temperatures. It also influences the fermentation efficiency, and actively participates in the flocculation process of yeast [51]. This element is extremely important for the activity of key enzymes in the beer production process, i.e., amylolytic and proteolytic enzymes. Sodium can affect the sensory properties of beer beyond a certain threshold of 150 mg/L resulting in a sour and salty flavor. The optimal levels of this metal are in the range of 75–150 mg/L, which contributes to round smoothness and emphasized sweetness [52]. Minerals such as zinc, and magnesium are necessary to obtain appropriate efficiency of the alcoholic fermentation as they act as cofactors of many yeast enzymes, and participate in the regulation of metabolic changes in yeast cells [53]. The zinc level in the wort is especially important in the brewing industry as it is one of the nutrients that can be deficient. Zinc plays an important role in fermentative metabolism, as it is essential for the alcohol dehydrogenase activity, and stimulates the intake of fermentable sugars like maltose and maltotriose. Zinc has also a positive effect on the viability of yeast cells [54] and is necessary for protein synthesis. Zinc deficiency (less than 0.1 mg/L) in the wort can lead to poor proliferation of yeast cells, incomplete diacetyl reduction, and slow fermentation [55].

In the wort samples made of barley malt, 4.63–5.7 mg/L of Ca^2+^ ions were determined (Table 2). In all worts obtained with the use of lentils grains significantly higher content of calcium ions was found. All samples were within the range 9.8–18.6 mg/L. The addition of proteolytic and amylolytic enzymes did not change the concentration of calcium ions in the analyzed samples. The lentils contained higher levels of calcium ions than barley, but these differences are not that significant in practical terms (Table 3). Values from 10 to 60 mg/L of Ca^2+^ have been reported in the literature in congress wort [45].

Sodium (Na^+^) levels in worts obtained from grains without enzymes addition were at a similar level of 9.4–12.7 mg/L. The addition of amylolytic enzymes caused a significant increase in Na^+^. Contents between 106 and 109 mg/L were found in all the samples with α-amylase. Proteolytic breakdown due to protease enzyme addition did not have the same effect. Wort from lentil (raw and malted) contained 9.4–12.7 of Na^+^, levels similar to barley malt. The results clearly show that α-amylase addition might result in highly elevated levels of sodium in a brewer’s wort, both when lentils and barley malts are used. Therefore, the content of these ions should be monitored when α-amylase is used in a brewing process.

In the wort produced from barley malt, 0.44 mg/L Zn^2+^ was determined. The content of zinc in barley malt was 2.3 mg/100 g. d.m. Worts made from both raw and malted green lentils had higher zinc content (1.42 mg/L and 1.8 mg/L, respectively) compared to the reference. This is due to the higher zinc content in lentil (4.2 mg/100 g d.m.) compared to barley (2.6 mg/100 g d.m). Additionally, it seems that zinc ions are easily available, soluble in water, and do not need to be released from the grain matrix in order to be presented in the wort. Interestingly, the addition of enzymes to the mash resulted in a reduction of the zinc ions content which can be seen in all of the samples to which enzymes were added. The minimum level of zinc available in the wort for yeast to achieve maximum fermentation yield is between 0.1 and 0.15 mg/L of Zn^2+^ [55,56]. However, higher concentrations of up to 0.9 mg/L have been reported [57]. Zn^2+^ values in worts made from green lentils were higher than recommended. However, it should be mentioned that the given values refer to the first wort, and during the further stages of the production of the wort, e.g., boiling and clarification, this amount is reduced by precipitation with hot trubs. This is important information for brewers and technologists, suggesting that green lentil malt can be a supplement added during wort production in low-zinc worts made from raw materials such as rice or corn to increase its final content in pitching wort.

The content of magnesium in barley grain was 92.4 mg/100 g d.m., while in barley malt was 95.1 mg/100 g. d.m. Malting of barley grain did not change the content of magnesium ions. However, malting reduced the magnesium ion content in lentils (Table 3).

#### 3.1.11. Carbohydrate Profile

Table 4 shows the concentration of sugars in the congress wort produced using various types of grist. Barley contains 0.03 to 0.6 g of glucose per 100 g of dry matter [25], while in malted grain, it is 2.2 g per 100 g of dry matter [52]. Lentil grains do not contain glucose in their carbohydrate composition, or they are detected at a very low level—up to 0.04 g of glucose per 100 g [58]. This is why worts made from green lentil malt and green lentils differ in carbohydrate profile when compared to barley malt wort (BM).

The highest glucose content was found in wort made from barley malt (6.07 g/L), which aligns with the literature stating that congress wort made from barley malt contains 5 to 7 g of glucose per liter of wort [25]. No glucose was found in the wort made from green lentil malt (ML), while in samples from raw green lentils (RL), the glucose content was 0.2 g/L. This changed when an enzyme with α-amylase activity was added to the mash, increasing the glucose content in wort made from raw lentils to 4.55 g/L and from malted lentils to 5.3 g/L. This enzyme liquefies the starch found in green lentils, breaking it down into simpler sugars, such as glucose, maltose, and maltotriose, which are then more accessible to the naturally occurring amylolytic enzymes in the grain. Lentil grains do not contain glucose in their carbohydrate composition, or they are detected at a low level of detection—up to 0.04 g of glucose per 100 g [16,59].

In the case of fructose, the results differed. The highest fructose concentration was found in wort from green lentil malt (1.31 g/L), compared to 0.92 g/L in barley malt. Enzyme addition increased fructose in barley malt to 1.26 g/L. Although Duke & Henson [60] indicate that barley malt provides more sucrose, lentil malt still offers significant fructose levels, especially after malting. This makes lentil malt a promising option for sour beer production, as it can effectively contribute to the natural acidification process by providing sufficient fructose for lactic acid fermentation. This information is crucial for future research, as it suggests that incorporating lentil malt into the grist could enhance the natural acidification of the wort.

The concentration of disaccharides (maltose and sucrose) was measured in various samples. The highest concentration was observed in the barley malt sample, which had a disaccharide concentration of 44 g/L. In contrast, the raw lentil (RL) and malted lentil (ML) samples exhibited relatively low disaccharide concentrations of 3.85 g/L and 6.57 g/L, respectively. Malting the lentil grains increases maltose and sucrose levels in the resultant wort. The addition of enzymes did not alter the disaccharide content in the malted barley samples. However, enzyme treatment had a notable effect on the lentil samples. A significant rise in disaccharide concentration was observed with the application of amylolytic enzymes, with ML + α-amylase achieving 16.34 g/L and RL + α-amylase reaching 11.70 g/L. Conversely, the application of protease enzymes did not improve disaccharide concentrations and actually reduced them. This indicates that the endogenous amylolytic activity of lentils is insufficient for complete saccharification of starch and that the addition of amylolytic enzymes is necessary.

The total sugar content is a key indicator of the fermentative potential of the obtained worts. Unlike the barley malt (BM) sample, both raw lentil (RL) and malted lentil (ML) samples contained relatively low amounts of fermentable sugars. Although these levels are not ideal for producing beers with standard alcohol content, lentil malt may be suitable for brewing low-alcohol beers. The addition of amylolytic enzymes led to a nearly fourfold increase in sugar levels compared to the base samples, thereby enhancing the potential for producing beers with higher alcohol content than what is achievable with the base raw materials.

## 4. Conclusions

The aim of this study was to produce brewer’s wort from malted green lentils and examine its biochemical characteristics in comparison to reference barley malt wort. The results showed that, compared to the reference wort (100% barley malt), the wort produced from malted lentils did not undergo saccharification, had a significantly longer filtration time (seven times longer), and exhibited lower extract content. The lentil malt wort had a darker color and significantly less turbidity.

A notable advantage of using lentil malt is its almost twofold higher content of free amino nitrogen (FAN) and total nitrogen, as well as nearly double the amount of zinc and calcium ions compared to barley malt. These factors are beneficial for yeast growth and fermentation efficiency. Additionally, lentils provide health and dietary benefits, as they are gluten-free and rich in essential micronutrients like zinc, magnesium, and potassium, along with vitamins such as B9 and B1.

Lentils contribute unique sensory attributes, including a richer flavor profile and fuller body, which could be particularly advantageous in the production of low-alcohol and sour beers. The high protein content of lentils enhances the body and mouthfeel of these beers, effectively compensating for the reduced fermentable sugars typically found in low-alcohol formulations.

Despite having a higher protein content compared to barley, proper management of the technological process, especially the use of enzymes (α-amylase), enables the achievement of favorable quality parameters in lentil-based wort. In this study, the addition of α-amylase to the mash with lentil malt resulted in saccharification and a significant increase in extract concentration in the wort, compared to the sample without the enzyme.

In conclusion, lentils present a promising and versatile ingredient for beer production, enhancing both the nutritional and sensory qualities of the final product. Their ability to increase FAN levels and provide unique flavor characteristics makes them an attractive option for brewers looking to innovate and diversify their product offerings. While lentils may not fully replace barley malt, they can be effectively utilized as a partial substitute, enhancing specific beer characteristics.

From an economic perspective, incorporating lentils into brewing processes may provide cost-effective advantages, particularly for brewers seeking to diversify their products. The unique qualities of lentil malt may appeal to health-conscious consumers, creating new market opportunities. However, further economic analysis is needed to assess the feasibility of large-scale production.

Future research should focus on optimizing the malting and brewing processes for lentil grains, particularly examining the biochemical interactions that influence the final product. This includes comprehensive sensory evaluations to refine flavor profiles and overall quality. Additionally, exploring various enzyme applications and malt combinations may further enhance the brewing potential of lentils. Overall, lentils represent a versatile and innovative ingredient for brewers seeking to explore new textures, flavors, and nutritional profiles, particularly in the development of gluten-free alternatives.

## Figures and Tables

**Table 1:** Physicochemical characteristics of worts and worts with added enzymes.

Parameter	BM	RL	ML	BM + A	RL + A	ML + A	BM + P	RL + P	ML + P
Saccharification time (min)	5–10	x	x	5–10	40	25–30	5–10	x	x
Filtration time (min/100 mL)	10.7 a (±11.5)	50.0 abcd (±5.00)	80.0 cd (±15.0)	8.00 a (±2.00)	25.3 ab (±4.62)	64.0 bcd (±36.1)	28.0 ab (±2.00)	95.7 d (±30.1)	42.7 abc (±8.33)
pH	5.89 (±0.04)	6.41 (±0.02)	6.05 (±0.05)	5.85 (±0.04)	6.35 (±0.04)	6.14 (±0.09)	5.88 (±0.04)	6.32 (±0.03)	6.03 (±0.09)
Color (EBC)	5.05 b (±0.25)	8.95 a (±0.25)	37.8 c (±3.61)	5.20 b (±0.67)	11.5 a (±0.60)	40.2 cd (±0.00)	8.30 ab (±0.72)	10.4 a (±0.60)	43.6 d (±0.60)
Extract (°P)	9.00 b (±0.30)	2.73 c (±0.06)	3.37 ac (±0.32)	9.13 b (±0.38)	6.53 d (±0.06)	6.67 d (±0.06)	9.27 b (±0.29)	3.57 a (±0.31)	3.63 a (±0.06)
Turbidity (NTU)	84.0 d (±4.00)	41.0 bc (±5.00)	16.4 ab (±1.45)	6.37 a (±1.58)	6.00 a (±1.00)	16.5 ab (±5.68)	50.0 c (±25.0)	5.85 a (±0.85)	24.2 ab (±0.15)
Polyphenols (mg/L)	86.5 a (±6.55)	188 bc (±15.9)	125 ab (±26.7)	81.9 a (±17.9)	228 cd (±10.4)	99.3 a (±22.4)	114 a (±18.1)	285 d (±49.4)	121 a (±6.00)
Free Amino Nitrogen (mg/L)	157 ab (±5.82)	172 ab (±4.12)	311 cd (±26.7)	143 a (±3.2)	166 ab (±29.0)	280 ce (±22.6)	219 be (±12.1)	309 cd (±36.8)	352 d (±38.4)
Total nitrogen (mg/L)	693 a (±6.11)	1219 d (±11.4)	1557 b (±35.1)	753 a (±15.3)	1313 e (±36.4)	1533 b (±31.6)	1073 c (±20.5)	1692 f (±22.2)	2446 g (±35.2)

(BM—100% barley malt; RL—100% raw lentils; ML—100% malted lentils; RL + A—100% raw lentils with the addition of α-amylase enzyme; ML + A—100% malted lentils with the addition of the enzyme α-amylase; RL + P—100% raw lentils with the addition of the protease enzyme; ML + P—100% malted lentils with the addition of the protease enzyme). The obtained values were statistically compared using multivariate analysis of variance—ANOVA. The results of statistical significance correspond to the interaction of all variables. a–g—mean values marked with different letters (for individual parameters) show statistical variation according to Tukey’s test (HSD) (*p* < 0.05).

**Table 2:** Metal ions content in worts without and with the addition of enzymes.

Metal Ions	BM	RL	ML	BM + A	RL + A	ML + A	BM + P	RL + P	ML + P
Mg^2+^ (mg/L)	76.3 (±0.87)	92.7 (±7.22)	87.9 (±16.3)	67.6 (±3.73)	79.4 (±7.37)	81.0 (±6.99)	69.0 (±2.74)	90.2 (±12.1)	90.2 (±9.62)
Zn^2+^ (mg/L)	0.44 (±0.16)	1.42 (±0.30)	1.80 (±0.71)	0.00 (±0.00)	0.70 (±0.10)	1.54 (±0.14)	0.00 (±0.00)	0.46 (±0.21)	1.07 (±0.28)
Ca^2+^ (mg/L)	4.63 (±1.79)	11.8 (±2.02)	18.6 (±4.33)	5.70 (±3.01)	9.80 (±0.82)	13.0 (±2.50)	5.70 (±1.02)	11.7 (±1.40)	14.1 (±1.66)
Na^+^ (mg/L)	10.2 (±1.66)	9.4 (±1.17)	12.7 (±1.80)	107 (±15.23)	106 (±2.66)	109 (±6.25)	15.0 (±3.73)	11.9 (±1.97)	12.2 (±2.47)

(BM—100% barley malt; RL—100% raw lentils; ML—100% malted lentils; RL + A—100% raw lentils with the addition of α-amylase enzyme; ML + A—100% malted lentils with the addition of the enzyme α-amylase; RL + P—100% raw lentils with the addition of the protease enzyme; ML + P—100% malted lentils with the addition of the protease enzyme). The obtained values were statistically compared using multivariate analysis of variance—ANOVA. The results of statistical significance correspond to the interaction of all variables. No assigned letters indicate the absence of statistically significant differences in each group of results at *p* < 0.05.

**Table 3:** Content of metal ions in grains.

Metal Ions	Raw Barley	Malted Barley	Raw Lentil	Malted Lentil
Mg^2+^ (mg/L)	92.4 a (±0.75)	95.1 ab (±4.30)	101 b (±1.33)	92.9 a (±2.42)
Zn^2+^ (mg/L)	2.63 a (±0.55)	2.33 a (±0.06)	4.17 b (±0.21)	4.67 b (±0.25)
Ca^2+^ (mg/L)	9.93 b (±0.25)	8.30 a (±0.55)	11.9 c (±0.06)	14.8 d (±0.15)

The obtained values were statistically compared using one-way analysis of variance—ANOVA. a–d—mean values marked with different letters (for individual parameters) show statistical variation according to Tukey’s test (HSD) (*p* < 0.05).

**Table 4:** Sugar content in worts with and without enzymes.

Parameter	BM	RL	ML	BM + α-Amylase	RL + α-Amylase	ML + α-Amylase	BM + Protease	RL + Protease	ML + Protease
Glucose (g/L)	6.07 e (±0.05)	0.20 ab (±0.00)	0.00 a (±0.00)	9.89 g (±0.11)	4.55 c (±0.09)	5.30 d (±0.02)	8.45 f (±0.08)	0.15 ab (±0.03)	0.27 b (±0.15)
Fructose (g/L)	0.92 b (±0.02)	0.52 a (±0.01)	1.31 c (±0.14)	1.26 c (±0.15)	0.58 a (±0.05)	1.26 c (±0.08)	1.03 b (±0.07)	0.49 a (±0.02)	0.96 b (±0.05)
Sucrose + Maltose (g/L)	44.0 a (±0.49)	3.85 c (±0.22)	6.57 e (±0.14)	41.9 h (±0.30)	11.7 f (±0.20)	16.3 g (±0.29)	43.6 a (±0.56)	2.84 b (±0.45)	5.37 d (±0.29)
Total sugars (g/L)	51.0 g (±0.52)	4.57 a (±0.24)	7.88 d (±0.28)	53.0 b (±0.44)	16.8 e (±0.17)	22.9 f (±0.31)	53.0 b (±0.65)	3.48 a (±0.49)	6.59 c (±0.40)

(BM—100% barley malt; RL—100% raw lentils; ML—100% malted lentils; RL + α-amylase—100% raw lentils with the addition of α-amylase enzyme; ML + α—amylase—100% malted lentils with the addition of the enzyme α-amylase; RL + protease—100% raw lentils with the addition of the protease enzyme; ML + protease—100% malted lentils with the addition of the protease enzyme). The obtained values were statistically compared using multivariate analysis of variance—ANOVA. The results of statistical significance correspond to the interaction of all variables. a-h—mean values marked with different letters (for individual parameters) show statistical variation according to Tukey’s test (HSD) (*p* < 0.05).

## Data Availability

The original contributions presented in this study are included in the article. Further inquiries can be directed to the corresponding author.
